# Supplementary material for: Long-term transverse imaging of the hippocampus with glass microperiscopes
Source: eLife. 2022 Jul 1;11:e75391. doi: 10.7554/eLife.75391 (PMC9249394; doi:10.7554/eLife.75391)
Supplement: Transparent reporting form [file elife-75391-transrepform1.pdf]

Westbrook Centre
Milton Road
Cambridge CB4 1YG, UK
P 01223 855340
W elifesciences.org
T @elife

eLife's transparent reporting form

We encourage authors to provide detailed information *within their submission* to facilitate the interpretation and replication of experiments. Authors can upload supporting documentation to indicate the use of appropriate reporting guidelines for health-related research (see EQUATOR Network), life science research (see the BioSharing Information Resource), or the ARRIVE guidelines for reporting work involving animal research. Where applicable, authors should refer to any relevant reporting standards documents in this form.

If you have any questions, please consult our Journal Policies and/or contact us: editorial@elifesciences.org.

Sample-size estimation

- You should state whether an appropriate sample size was computed when the study was being designed
- You should state the statistical method of sample size computation and any required assumptions
- If no explicit power analysis was used, you should describe how you decided what sample (replicate) size (number) to use

Please outline where this information can be found within the submission (e.g., sections or figure legends), or explain why this information doesn't apply to your submission:

All numbers pertaining to sample-size are reported in the main text, figure captions, and supplementary material. Previously published work was used to determine the number of mice and neurons/spines appropriate for our experiments. In particular:

1. We tracked spines in a similar number of mice as Pfeiffer et al., 2018. and Attardo et al., 2015, with as equal or greater dendritic segments imaged than both prior works.

2. We recorded from a similar number of active neurons in each hippocampal subfield as Hainmueller and Bartos, 2018. Since they were restricted to a single subfield per mouse, they used more mice total than we did, although we had a similar number of mice per subfield.

3. We recorded a similar number of active neurons along the CA1-DG axis as Lu et al., 2015, although using a significantly fewer number of mice. This is because we could image along the extent of the axis in each mouse, whereas they had to sample from more mice to attain the same coverage.

For the microsphere optical characterization experiments, we used only clearly isolated microspheres (nearest identified microsphere >20 pixels away). Since the standard deviations of the fluorescence measurements were small, we determined our sample (10-20 microspheres) to be sufficient.

For the histology experiments, we used fewer mice and slices than previous experiments (e.g., Andermann et al, 2013). However, since the standard error estimates for our measurements was small, we considered our sample sufficient.

Replicates

- You should report how often each experiment was performed
- You should include a definition of biological versus technical replication
- The data obtained should be provided and sufficient information should be provided to indicate the number of independent biological and/or technical replicates
- If you encountered any outliers, you should describe how these were handled
- Criteria for exclusion/inclusion of data should be clearly stated
- High-throughput sequence data should be uploaded before submission, with a private link for reviewers provided (these are available from both GEO and ArrayExpress)

Please outline where this information can be found within the submission (e.g., sections or figure legends), or explain why this information doesn't apply to your submission:

> For all biological experiments, we repeated the experiments in multiple mice. The number of mice (biological replicates) are reported in the main text, figure captions, and supplementary material.

Statistical reporting

- Statistical analysis methods should be described and justified
- Raw data should be presented in figures whenever informative to do so (typically when N per group is less than 10)
- For each experiment, you should identify the statistical tests used, exact values of N, definitions of center, methods of multiple test correction, and dispersion and precision measures (e.g., mean, median, SD, SEM, confidence intervals; and, for the major substantive results, a measure of effect size (e.g., Pearson's r, Cohen's d)
- Report exact p-values wherever possible alongside the summary statistics and 95% confidence intervals. These should be reported for all key questions and not only when the p-value is less than 0.05.

Please outline where this information can be found within the submission (e.g., sections or figure legends), or explain why this information doesn't apply to your submission:

> All means with their precision measures (standard deviations, standard error, or confidence interval) are reported in the main text and figure captions. All exact p-values are reported in the main text and figure captions. All statistical test are reported in the main text and figure captions.
>
> Violin plots were used for plotting whenever possible. In the case of plotting place cell responses, cross-validated place fields were displayed for transparency.

(For large datasets, or papers with a very large number of statistical tests, you may upload a single table file with tests, Ns, etc., with reference to sections in the manuscript.)

Group allocation

- Indicate how samples were allocated into experimental groups (in the case of clinical studies, please specify allocation to treatment method); if randomization was used, please also state if restricted randomization was applied

- Indicate if masking was used during group allocation, data collection and/or data analysis

Please outline where this information can be found within the submission (e.g., sections or figure legends), or explain why this information doesn't apply to your submission:

Not applicable. No group allocation was performed in this paper since all comparisons were within-animal.

Additional data files ("source data")

- We encourage you to upload relevant additional data files, such as numerical data that are represented as a graph in a figure, or as a summary table
- Where provided, these should be in the most useful format, and they can be uploaded as "Source data" files linked to a main figure or table
- Include model definition files including the full list of parameters used
- Include code used for data analysis (e.g., R, MatLab)
- Avoid stating that data files are "available upon request"

Please indicate the figures or tables for which source data files have been provided:

As described in the data availability statement, all data and code will be made available on public archives upon acceptance of the paper.
